# Co-circulation of diverse paramyxoviruses in an urban African fruit bat population

**DOI:** 10.1099/vir.0.039339-0

**Published:** 2012-04

**Authors:** K. S. Baker, S. Todd, G. Marsh, A. Fernandez-Loras, R. Suu-Ire, J. L. N. Wood, L. F. Wang, P. R. Murcia, A. A. Cunningham

**Affiliations:** 1 Institute of Zoology, Zoological Society of London, Regent’s Park, London NW1 4RY, UK; 2 Cambridge Infectious Diseases Consortium, University of Cambridge, Department of Veterinary Medicine, Madingley Road, Cambridge CB3 0ES, UK; 3 CSIRO Australian Animal Health Laboratories, Portarlington Road, East Geelong, VIC 3219, Australia; 4 Wildlife Division of the Forestry Commission, Accra, Ghana; 5 University of Glasgow Centre for Virus Research, Institute of Infection, Immunity and Inflammation, College of Medical, Veterinary and Life Sciences, Garscube Estate, Bearsden Road, Glasgow G61 1QH, UK

## Abstract

Bats constitute a reservoir of zoonotic infections and some bat paramyxoviruses are capable of cross-species transmission, often with fatal consequences. Determining the level of viral diversity in reservoir populations is fundamental to understanding and predicting viral emergence. This is particularly relevant for RNA viruses where the adaptive mutations required for cross-species transmission can be present in the reservoir host. We report the use of non-invasively collected, pooled, neat urine samples as a robust sample type for investigating paramyxoviruses in bat populations. Using consensus PCR assays we have detected a high incidence and genetic diversity of novel paramyxoviruses in an urban fruit bat population over a short period of time. This may suggest a similarly unique relationship between bats and the members of the family *Paramyxoviridae* as proposed for some other viral families. Additionally, the high rate of bat–human contact at the study site calls for the zoonotic potential of the detected viruses to be investigated further.

Bats are an increasingly recognized source of emerging zoonoses and are known to harbour a wide range of viruses, including highly virulent zoonoses such as henipa-, filo- and lyssa- viruses (Calisher *et al.*, 2006). Consequently, research into bat viromes is intensifying and results obtained from those investigations show that viruses in bat populations exhibit significant genetic diversity. Marked diversity of lyssaviruses (Delmas *et al.*, 2008), coronaviruses (Tang *et al.*, 2006; Woo *et al.*, 2006), astroviruses (Chu *et al.*, 2008) and, more recently, adenoviruses (Li *et al.*, 2010) and circoviruses (Ge *et al.*, 2011) has been reported. In some cases, this diversity has led to speculation that chiropterans have ancient relationships with these viral families, and consequently act as reservoirs for emergence (Badrane & Tordo, 2001; Gouilh *et al.*, 2011). Bats are known to harbour multiple paramyxoviruses, including rubulaviruses and henipaviruses that have spilled over into humans and/or domestic animals (Calisher *et al.*, 2006; Chant *et al.*, 1998; Lau *et al.*, 2010).

African straw-coloured fruit bats (*Eidolon helvum*) sampled in Ghana have been shown to have neutralizing antibodies against henipaviruses (Hayman *et al.*, 2008) as well as paramyxoviral RNA in their faeces (Drexler *et al.*, 2009). Paramyxoviruses are known to be excreted in the urine of experimentally infected and wild fruit bats (Middleton *et al.*, 2007; Chua *et al.*, 2002; Smith *et al.*, 2011). Here, we report the paramyxovirus diversity in neat urine samples collected from underneath a large population of *E. helvum* in Accra, Ghana, which lives in close proximity to humans and domestic animals.

Pooled urine samples (*n* = 72) from *E. helvum* were collected over 12 sampling intervals between September and November 2010 (Table 1). Samples were collected from underneath a tree holding ~1500 *E. helvum* (co-roosting with other species has not been observed at this site) that comprise part of a much larger colony of up to 1 000 000 bats in Accra (Hayman *et al.*, 2008). Plastic sheeting was laid out underneath the tree approximately 1 h before dawn (adapted from Chua, 2003). On return from foraging, bats urinated and defecated on the sheets and 1 ml pools of neat urine were collected by pipette and divided equally between two vials for PCR analysis and virus isolation. Care was taken to avoid contact with faeces but it is possible that small amounts of faecal contamination were present in the samples. Samples were stored and transported at ~4 °C before freezing and stored at −80 °C until further processing. RNA was extracted from 500 µl neat urine from each pool using the MagMAX viral RNA isolation kit and a MagMAX Express-96 automated extraction unit (Life Technologies – Applied Biosystems), using a 1 : 2 ratio of sample to lysis buffer.

**Table 1.** Collection dates, PCR results and clone notations of pooled urine samples used in this study

Date	Sample ID	PMV-PCR	Clone(s)*	RMH-PCR	Clone(s)*
24/09/2010	U1				
	U2				
	U3				
	U4				
	U5	+	B, D	+	A, B, C
	U6	+	A, B	+	A, B
30/09/2010	U7				
	U8				
	U9	+	B, D		A
	U10				
	U11				
04/10/2010	U12				
	U13				
	U14				
	U15				
	U16				
08/10/2010	U17				
	U18				
	U19				
	U20				
12/10/2010	U21				
	U22	+	C		
	U23				
	U24				
	U25				
	U26				
14/10/2010	U27				
	U28				
	U29				
	U30				
	U31				
19/10/2010	U32	+	C		
	U33				
21/10/2010	U34				
	U35				
	U36				
	U37				
	U38				
02/11/2010	U39				
	U40				
	U41				
	U42	+	A, B	+	A, B
10/11/2010	U43	+	B		
	U44	+	A, B		
	U45			+	A, B
	U46	+	B, G, H		
	U47	+	C, E, F, G, H		
	U48				
	U49	+	B	+	B
	U50	+	A, B	+	B, C
15/11/2010	U51			+	A, B
	U52	+	B, C		
	U53	+	A, C	+	A, B
	U54	+	C, D	+	A, B
	U55	+	C		
	U56				
	U57	+	B, C		
	U58			+	B
	U59	+	C	+	A, B
	U60				
	U61			+	A, B
	U62			+	A, B
20/11/2010	U63			+	A, B
	U64	+	A	+	A, B
	U65				
	U66	+	A	+	A, B
	U67	+	J, N	+	A
	U68	+	E, G	+	A, B
	U69	+	C, D		
	U70				
	U71	+	C	+	A, B
	U72			+	A, B
**Total**	**Samples**	**Positive**	**PMV-PCR clones**	**Positive**	**RMH-PCR clones**
	72	24	43	21	39

* Individual cloned sequences from PCR products are noted by individual letters.

Extracted RNA was tested for the presence of paramyxovirus polymerase gene RNA using two heminested RT-PCRs (Tong *et al.*, 2008). *Paramyxovirinae* PCR (PMV-PCR) was used to amplify a 531 bp (excluding primers PAR-F2 and PAR-R) fragment of polymerase genes of viruses belonging to the subfamily *Paramyxovirinae*, that spanned positions 13 898–14 428 of the hendra virus genome (GenBank accession no. NC_001906). A PCR targeting an upstream portion of the polymerase gene (~439 bp, excluding primers RES-MOR-HEN-F2 and RES-MOR-HEN-R, corresponding to positions 12 617–13 055 of the hendra virus genome) of respiro-, morbilli- and henipa- viruses (RMH-PCR) was also used.

PCR products were cloned into pGEM-T Easy (Promega) and one or more clones from each sample were sequenced (Big Dye Terminator v3.1; Life technologies – Applied Biosystems). Obtained sequences were aligned with polymerase genes from the subfamily *Paramyxovirinae* using muscle (Edgar, 2004) and clustal_x (Thompson *et al.*, 1994). Phylogenetic trees were constructed using Mr Bayes (Ronquist & Huelsenbeck, 2003) under the GTR+I+G model (as determined by modeltest; Posada & Crandall, 1998).

The urine samples were frequently found to contain paramyxovirus sequences with PCR-positive samples being collected on 8 of the 12 sampling dates (over a 2 month period) and 31 of 72 samples (43 %) being positive. Fifteen of the samples were positive by both PMV-PCR and RMH-PCR, with each PCR detecting a further 10 and 7 (respectively) positive samples uniquely (Table 1, Figs 1 and 2). Phylogenetic analysis of sequences derived from cloned PCR products from the PMV-PCR (43 clones) and RMH-PCR (39 clones) not only revealed the relationships of the novel sequences with each other and known sequences** from** the subfamily *Paramyxovirinae*, but also allowed for comparison of the assays’ performance on field samples (Figs 1 and 2). GenBank accession numbers for novel paramyxovirus sequences are shown in Figs 1 and 2.

**Fig. 1.**
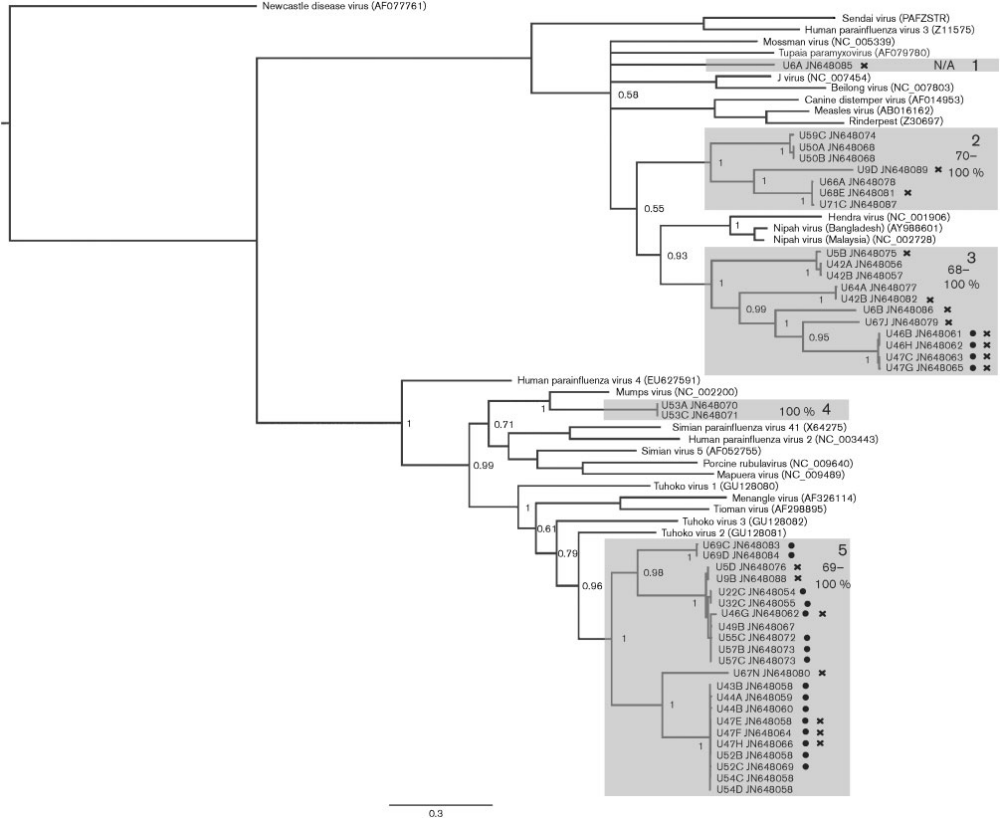
Diversity of paramyxoviruses in pooled *E. helvum* urine samples detected by using *Paramyxovirinae*-targeted PCR. Phylogenetic tree for a 531 bp segment of the polymerase gene of members of the subfamily *Paramyxovirinae*, including sequences generated in this study and publicly available paramyxovirus sequences (Newcastle disease virus, sendai virus, human parainfluenza virus 3, mossman virus, tupaia paramyxovirus, J virus, beilong virus, canine distemper virus, measles virus, rinderpest, hendra virus, nipah virus (Bangladesh), nipah virus (Malaysia), human parainfluenza virus 4, mumps virus, simian parainfluenza virus 41, human parainfluenza virus 2, simian virus 5, porcine rubulavirus, mapuera virus, tuhoko virus 1, menangle virus, tioman virus, tuhoko virus 3, tuhoko virus 2). Relevant posterior probability values are shown. Bar, 0.3 expected nucleotide substitutions per site. Individual sample IDs are followed by letters denoting the clone and the GenBank accession number for the sequence. Groups containing previously uncharacterized sequences that display a common phylogenetic origin supported by high posterior probability values (≥0.95) are highlighted by numbered grey boxes. Adjacent to each box number is the range of nucleotide identities among sequences in the clade. Clones derived from samples that contained sequences belonging to more than one of these phylogenetically distinct clades were marked with a cross. Sequences derived from samples that were positive only with this PCR (and negative with RMH-PCR) are marked with a dot.

**Fig. 2.**
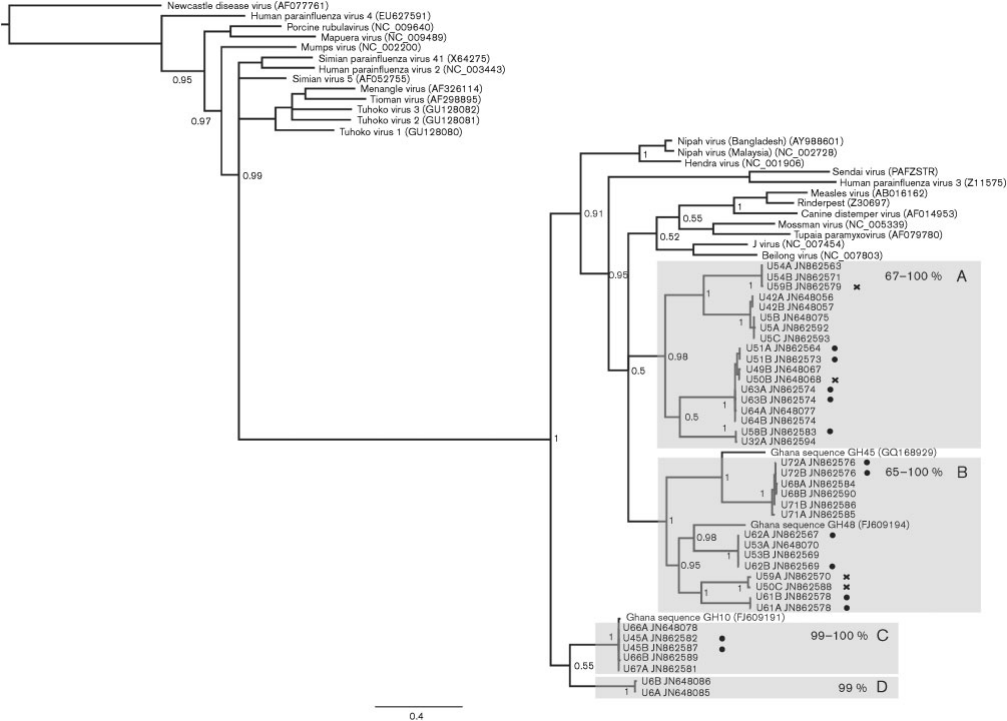
Diversity of paramyxoviruses in pooled *E. helvum* urine samples detected by using RMH-targeted PCR. Phylogenetic tree for a 439 bp gap-stripped alignment of the polymerase gene of members of the subfamily *Paramyxovirinae*, including sequences generated in this study and publicly available paramyxovirus genome sequences (as per Fig. 1) and partial paramyxovirus sequences available from this species (Ghanaian sequences GH45, GH48 and GH10). Relevant posterior probability values are shown. Bar, 0.4 expected nucleotide substitutions per site. Individual sample IDs are followed by letters denoting the clone and the GenBank accession for the sequence. Groups containing previously uncharacterized sequences that display a common phylogenetic origin supported by high posterior probability values (≥0.95) are highlighted by lettered grey boxes. Adjacent to each box letter is the range of nucleotide identities among sequences in the clade. Clones derived from samples that contained sequences belonging to more than one of these phylogenetically distinct clades were marked with a cross. Sequences derived from samples that were positive only with this PCR (and negative with PMV*-*PCR) are marked with a dot.

The sequences obtained through PCR indicated the presence of multiple novel paramyxoviruses circulating in the *E. helvum* population in Accra, with sequences having pairwise nucleotide identities of 57–89 % with known paramyxoviruses. These sequences expanded the *Paramyxovirinae* phylogeny, with the PMV-PCR detecting sequences in five clusters throughout the phylogeny (Fig. 1). Some clusters (2, 3 and 5 in Fig. 1) represented phylogenetically diverse subgroups that shared a common ancestor supported by high posterior probability values, and two of these clusters (1 and 2, Fig. 1) did not group confidently with any existing genus in the subfamily *Paramyxovirinae.* Another group of sequences detected by PMV-PCR were phylogenetically related to other unclassified bat-derived paramyxoviruses (menangle, tioman and tuhoko viruses) within the clade containing known rubulaviruses (5, Fig. 1).

Notably, some sequences were related to known human pathogens, including sequences derived from sample U53 using the PMV-PCR that grouped with mumps virus with a high posterior probability value (4, Fig. 1) and highly structured groups of sequences related to, but distinct from, henipaviruses (3, Fig. 1 and C and D, Fig. 2).

The sequences obtained using the RMH-PCR were, as expected, restricted to the portion of the *Paramyxovirinae* phylogeny pertaining to these genera (Fig. 2). However, consistent with the results obtained using the PMV-PCR, the sequences detected using the RMH-PCR similarly expanded this portion of the phylogeny, with sequences being distributed in four main clusters (A–D, Fig. 2). These clusters were related to, but distinct from, henipaviruses and respiroviruses but were otherwise unable to be confidently placed in existing genera of the *Paramyxovirinae* phylogeny. Clusters B and C (Fig. 2) were related to paramyxovirus sequences previously obtained from *E. helvum* faecal material in Ghana (Drexler *et al.*, 2009). However, some sequences in cluster B were still phylogenetically distinct from these (Fig. 2), sharing as little as 74 % nucleotide identity, perhaps suggesting that the sequences obtained thus far still represent only a fraction of the paramyxovirus diversity in *E. helvum*.

These results illustrate the high levels of paramyxovirus diversity in the sampled population, with the sequences detected by PMV-PCR having nucleotide identities as low as 42 % among them, and those detected through RMH-PCR as low as 57 %. Even subsequent to phylogenetic grouping, the nucleotide identities of sequences within the same clades were as low as ~65 % (e.g. 3, Fig. 1 and B, Fig. 2). Notably, individual sample pools were also genetically diverse. PCR products (where >1 clone was sequenced) often contained phylogenetically distinct sequences. For example, sequences U9B and U9D generated by PMV-PCR belonged to phylogenetic groups 5 and 2, respectively (see Fig. 1, and other sequences marked with crosses in Figs 1 and 2). This phenomenon was more commonly observed in samples using PMV-PCR (7/15) than those using RMH-PCR (2/17).

Here, we have detected a high incidence and diversity of paramyxoviruses in *E. helvum* in Accra, Ghana, using previously published consensus PCR assays together with sequencing of cloned PCR products. Both the PMV-PCR and RMH-PCR assays were highly effective at detecting novel paramyxovirus sequences and consequently will likely continue to be used in other field studies. However, they were not equivocal in their results and these analyses provide important insights into their differential capabilities that are likely applicable across other species and sites. The assays differed in their specificities, with the PMV-PCR detecting sequences throughout the *Paramyxovirinae* phylogeny (with the notable exception of sequences closely related to avulaviruses) and the RMH-PCR being restricted to respiro-, morbilli- and henipa- viruses. This is likely caused by the greater redundancy of the PMV-PCR primers and is further evidenced by the results from 10 samples in which sequence was detected using PMV-PCR but not using RMH-PCR. The sequences derived from these 10 samples showed phylogenetic clustering (Fig. 1), with the majority within the genus *Rubulavirus*. The broader specificity of the PMV-PCR is also what likely allowed this assay to detect intrasample diversity more frequently than was observed with the RMH-PCR. It should be noted, however, that sequences derived from the same sample could be derived from different viruses and/or bats due to the nature of the sampling method.

Regarding assay sensitivity, although the PMV-PCR and RMH-PCR appear superficially to have similar sensitivities (24 vs 21 of 72 positives, respectively), the RMH-PCR detected seven samples as positive that were negative on PMV-PCR. However, the phylogenetic clustering of these samples’ sequences was not as marked as in the previous case (Fig. 2). Thus, it is likely that the uniquely detected samples in the case of the RMH-PCR are the result of increased assay sensitivity as reported in the original *in vitro* studies (Tong *et al.*, 2008). Due to differences in phylogenetic tree topology produced by different polymerase regions, and the differing sensitivity and specificity of the assays, it is difficult to comment on the relationships of sample sequences generated by different PCRs. However, sample U6 notably generated unique and poorly placed clusters in phylogenetic analysis of both regions.

Irrespective of assay differences, novel paramyxovirus sequences were detected at a high incidence in pooled urine samples collected from underneath a single roost-tree of *E. helvum* in Accra, Ghana. This demonstrates that neat urine pools are a robust sample type for investigation into paramyxoviruses in bat populations. Indeed, novel paramyxoviruses have since been isolated and characterized from this population (K. S. Baker and others, unpublished data). Not only is collecting urine from underneath roosts highly efficacious for qualitative virus population studies, but the non-invasive nature of the collection method would facilitate the study of infection dynamics free from sampling-induced stress. However, one disadvantage is that the viral sequences detected here cannot be absolutely confirmed as having been derived from *E. helvum*. Although co-roosting with other species has not previously been observed, and sheeting was supervised throughout the sampling period, detection of these sequences in samples directly collected from individual bats would be required to confirm their origin.

The detected sequences are highly novel and demonstrate significant genetic diversity of paramyxoviruses within a single bat population over a short period of time. Furthermore, their close relationships with previously detected paramyxovirus sequences in Ghanaian *E. helvum* (Drexler *et al.*, 2009) and sequences detected in urine collected from *E. helvum* roosts in Uganda and Tanzania (A. J. Peel and others, unpublished results) supports these findings and increases our understanding of the diversity of paramyxoviruses in Africa. Indeed, the sequences described here support the further expansion of the genus *Rubulavirus*, which appears to have commenced with the detection of other bat-derived paramyxoviruses namely menangle, tioman and tuhoko viruses. Also, our data give rise to numerous phylogenetically distinct, but poorly placed clusters of viral sequences, perhaps indicating that viruses belonging to novel genera may be circulating in the study population. Such diversity and expansion of the paramyxovirus phylogeny within a single population implies that adaptive mutations required for emergence may be present in the reservoir host, and also may suggest that a co-evolutionary relationship between *Chiroptera* and *Paramyxoviridae* exists, as has been proposed for other viral families (Badrane & Tordo, 2001; Gouilh *et al.*, 2011). The presence of intrasample diversity is intriguing and may reflect co-infection of an individual, or alternatively may have resulted from the pooling of co-circulating viruses from different bats. Further studies, such as examination of urine samples from individual bats would be required to differentiate between these possibilities.

Sequences were detected that had close relationships with known human pathogens. *E. helvum* is widely hunted in Ghana for bush meat (Kamins *et al.*, 2011) and the population under study has a high potential for both direct and indirect contact with human beings and domestic animals. This information, coupled with the knowledge that many bat paramyxoviruses are already known to be zoonotic, makes it imperative that our findings are investigated further.

## References

[r1] Badrane H. Tordo N. **(**2001**).** Host switching in lyssavirus history from the Chiroptera to the Carnivora orders. J Virol 75, 8096–8104 10.1128/JVI.75.17.8096-8104.2001 11483755 PMC115054

[r2] Calisher C. H. Childs J. E. Field H. E. Holmes K. V. Schountz T. **(**2006**).** Bats: important reservoir hosts of emerging viruses. Clin Microbiol Rev 19, 531–545 10.1128/CMR.00017-06 16847084 PMC1539106

[r3] Chant K. Chan R. Smith M. Dwyer D. E. Kirkland P. D. The NSW Expert Group **(**1998**).** Probable human infection with a newly described virus in the family Paramyxoviridae. Emerg Infect Dis 4, 273–275 10.3201/eid0402.980215 9621198 PMC2640130

[r4] Chu D. K. Poon L. L. Guan Y. Peiris J. S. **(**2008**).** Novel astroviruses in insectivorous bats. J Virol 82, 9107–9114 10.1128/JVI.00857-08 18550669 PMC2546893

[r5] Chua K. B. **(**2003**).** A novel approach for collecting samples from fruit bats for isolation of infectious agents. Microbes Infect 5, 487–490 10.1016/S1286-4579(03)00067-4 12758277

[r6] Chua K. B. Koh C. L. Hooi P. S. Wee K. F. Khong J. H. Chua B. H. Chan Y. P. Lim M. E. Lam S. K. **(**2002**).** Isolation of Nipah virus from Malaysian Island flying-foxes. Microbes Infect 4, 145–151 10.1016/S1286-4579(01)01522-2 11880045

[r7] Delmas O. Holmes E. C. Talbi C. Larrous F. Dacheux L. Bouchier C. Bourhy H. **(**2008**).** Genomic diversity and evolution of the lyssaviruses. PLoS ONE 3, e2057 10.1371/journal.pone.0002057 18446239 PMC2327259

[r8] Drexler J. F. Corman V. M. Gloza-Rausch F. Seebens A. Annan A. Ipsen A. Kruppa T. Müller M. A. Kalko E. K. **& other authors (**2009**).** Henipavirus RNA in African bats. PLoS ONE 4, e6367 10.1371/journal.pone.0006367 19636378 PMC2712088

[r9] Edgar R. C. **(**2004**).** muscle: multiple sequence alignment with high accuracy and high throughput. Nucleic Acids Res 32, 1792–1797 10.1093/nar/gkh340 15034147 PMC390337

[r10] Ge X. Li J. Peng C. Wu L. Yang X. Wu Y. Zhang Y. Shi Z. **(**2011**).** Genetic diversity of novel circular ssDNA viruses in bats in China. J Gen Virol 92, 2646–2653 10.1099/vir.0.034108-0 21795473

[r11] Gouilh M. A. Puechmaille S. J. Gonzalez J. P. Teeling E. Kittayapong P. Manuguerra J. C. **(**2011**).** *SARS-Coronavirus* ancestor’s foot-prints in South-East Asian bat colonies and the refuge theory. Infect Genet Evol 11, 1690–1702 10.1016/j.meegid.2011.06.021 21763784 PMC7106191

[r12] Hayman D. T. Suu-Ire R. Breed A. C. McEachern J. A. Wang L. Wood J. L. Cunningham A. A. **(**2008**).** Evidence of henipavirus infection in West African fruit bats. PLoS ONE 3, e2739 10.1371/journal.pone.0002739 18648649 PMC2453319

[r13] Kamins A. O. Restif O. Ntiamoa-Baidu Y. Suu-Ire R. Hayman D. T. S. Cunningham A. A. Wood J. L. N. Rowcliffe J. M. **(**2011**).** Uncovering the fruit bat bushmeat commodity chain and the true extent of fruit bat hunting in Ghana, West Africa. Biol Conserv 144, 3000–3008 22514356 10.1016/j.biocon.2011.09.003 PMC3323830

[r14] Lau S. K. Woo P. C. Wong B. H. Wong A. Y. Tsoi H. W. Wang M. Lee P. Xu H. Poon R. W. Guo R. **(**2010**).** Identification and complete genome analysis of three novel paramyxoviruses, tuhoko virus 1, 2 and 3, in fruit bats from China. Virology 404, 106–116 10.1016/j.virol.2010.03.049 20537670 PMC7111929

[r15] Li Y. Ge X. Zhang H. Zhou P. Zhu Y. Zhang Y. Yuan J. Wang L. F. Shi Z. **(**2010**).** Host range, prevalence, and genetic diversity of adenoviruses in bats. J Virol 84, 3889–3897 10.1128/JVI.02497-09 20089640 PMC2849498

[r16] Middleton D. J. Morrissy C. J. van der Heide B. M. Russell G. M. Braun M. A. Westbury H. A. Halpin K. Daniels P. W. **(**2007**).** Experimental nipah virus infection in pteropid bats (*Pteropus poliocephalus*). J Comp Pathol 136, 266–272 10.1016/j.jcpa.2007.03.002 17498518

[r17] Posada D. Crandall K. A. **(**1998**).** modeltest: testing the model of DNA substitution. Bioinformatics 14, 817–818 10.1093/bioinformatics/14.9.817 9918953

[r18] Ronquist F. Huelsenbeck J. P. **(**2003**).** MrBayes 3: Bayesian phylogenetic inference under mixed models. Bioinformatics 19, 1572–1574 10.1093/bioinformatics/btg180 12912839

[r19] Smith I. Broos A. de Jong C. Zeddeman A. Smith C. Smith G. Moore F. Barr J. Crameri G. **& other authors (**2011**).** Identifying hendra virus diversity in pteropid bats. PLoS ONE 6, e25275 10.1371/journal.pone.0025275 21980413 PMC3182206

[r20] Tang X. C. Zhang J. X. Zhang S. Y. Wang P. Fan X. H. Li L. F. Li G. Dong B. Q. Liu W. **& other authors (**2006**).** Prevalence and genetic diversity of coronaviruses in bats from China. J Virol 80, 7481–7490 10.1128/JVI.00697-06 16840328 PMC1563713

[r21] Thompson J. D. Higgins D. G. Gibson T. J. **(**1994**).** clustal w: improving the sensitivity of progressive multiple sequence alignment through sequence weighting, position-specific gap penalties and weight matrix choice. Nucleic Acids Res 22, 4673–4680 10.1093/nar/22.22.4673 7984417 PMC308517

[r22] Tong S. Chern S. W. Li Y. Pallansch M. A. Anderson L. J. **(**2008**).** Sensitive and broadly reactive reverse transcription-PCR assays to detect novel paramyxoviruses. J Clin Microbiol 46, 2652–2658 10.1128/JCM.00192-08 18579717 PMC2519498

[r23] Woo P. C. Lau S. K. Li K. S. Poon R. W. Wong B. H. Tsoi H. W. Yip B. C. Huang Y. Chan K. H. Yuen K. Y. **(**2006**).** Molecular diversity of coronaviruses in bats. Virology 351, 180–187 10.1016/j.virol.2006.02.041 16647731 PMC7111821

